# Special Issue: “Molecules against Alzheimer”

**DOI:** 10.3390/molecules21121736

**Published:** 2016-12-16

**Authors:** Michael Decker, Diego Muñoz-Torrero

**Affiliations:** 1 Pharmazeutische und Medizinische Chemie, Institut für Pharmazie und Lebensmittelchemie, Julius-Maximilians-Universität Würzburg, Am Hubland, 97074 Würzburg, Germany; 2 Laboratory of Pharmaceutical Chemistry, Faculty of Pharmacy and Food Sciences, and Institute of Biomedicine (IBUB), University of Barcelona, Av. Joan XXIII, 27-31, E-08028 Barcelona, Spain

**Keywords:** Alzheimer’s disease, drug discovery, rational design, hybrid molecules, multi-target-directed molecules, polypharmacology, imaging agents, neuroprotectants, neurogenic molecules

## Abstract

This Special Issue, entitled “Molecules against Alzheimer”, gathers a number of original articles, short communications, and review articles on recent research efforts toward the development of novel drug candidates, diagnostic agents and therapeutic approaches for Alzheimer’s disease (AD), the most prevalent neurodegenerative disorder and a leading cause of death worldwide. This Special Issue contains many interesting examples describing the design, synthesis, and pharmacological profiling of novel compounds that hit one or several key biological targets, such as cholinesterases, β-amyloid formation or aggregation, monoamine oxidase B, oxidative stress, biometal dyshomeostasis, mitochondrial dysfunction, serotonin and/or melatonin systems, the Wnt/β-catenin pathway, sigma receptors, nicotinamide phosphoribosyltransferase, or nuclear erythroid 2-related factor. The development of novel AD diagnostic agents based on tau protein imaging and the use of lithium or intranasal insulin for the prevention or the symptomatic treatment of AD is also covered in some articles of the Special Issue.

Alzheimer’s disease (AD) is the most common cause of dementia and the most prevalent neurodegenerative disorder. AD currently accounts for up to 75% of the nearly 47 million people that suffer currently from dementia and has an associated global cost that represents 1.09% of the global gross domestic product [1], thereby constituting a major health and economic issue worldwide.

To date, only four inhibitors of the enzyme acetylcholinesterase (AChE) and one glutamate *N*-methyl-d-aspartate (NMDA) receptor antagonist have been approved for the treatment of AD, with all of them being regarded as symptomatic agents that alleviate the cognitive and functional deficits for a limited time [2]. Huge economic and research efforts have been invested during the past two decades to unveil the mechanisms that underlie AD pathogenesis, to identify key biological targets and pathological events and to discover novel therapeutic strategies and disease-modifying drugs that can prevent, halt, or delay the progression of the disease, as well as novel biomarkers and diagnostic agents that enable an early therapeutic intervention with those drugs. Unfortunately, since the launch of the fifth anti-Alzheimer drug in 2003, the clinical development of more than 100 anti-Alzheimer drug candidates has been discontinued, in many cases in advanced phases, due to efficacy or safety issues, thereby making AD drug development one of the therapeutic areas with the highest attrition rates [3,4].

In this context and in the light of the highly increased prevalence expected for the upcoming decades according to the demographic projections, the discovery of effective drug candidates, diagnostic agents, and alternative therapeutic strategies against AD constitutes a dire need. This *Molecules* Special Issue, edited by Professor Michael Decker (Julius-Maximilians-Universität Würzburg) and Professor Diego Muñoz-Torrero (University of Barcelona), consists of 16 excellent original articles, short communications, and review articles that describe recent research in the pursuit of novel drug candidates, diagnostic agents and therapeutic approaches against AD.

One of the alternative therapeutic approaches that is currently gaining more acceptance is based on the conception of AD pathogenesis as a pathological network with several pathological events running in parallel instead of a cascade of consecutive events. The logical positioning that follows this conception of the disease is that the most realistic way to alter the natural course of AD should involve the simultaneous modulation of several key biological targets or pathological events of that network [5,6,7,8]. Indeed, most of the articles of this Special Issue are related to the development of multitarget anti-Alzheimer compounds.

A. Carotti, M. Catto, et al. report on the structural modification of the 2*H*-chromen-2-one scaffold to derive a novel class of multitarget AChE-monoamine oxidase B (MAO-B) inhibitors, endowed with additional protective effects against the insult of mitochondrial toxins oligomycin-A and rotenone to human neuroblastoma SH-SY5Y cells, and brain permeation without glycoprotein-p liability [9].

In the frame of the development of multipotent non-toxic derivatives of the anti-Alzheimer drug tacrine, a series of imidazopyranotacrines is described by J. Marco-Contelles, A. Belfaitah, L. Ismaili, et al. as nonhepatotoxic inhibitors of AChE with potent antioxidant activity [10].

Three series of compounds featuring an isoindoline-1,3-dione, indole or benzo[*d*]isothiazol-3(2*H*)-one 1,1-dioxide moiety have been designed and synthesized by B. Malawska et al. as inhibitors of AChE with potential to simultaneously interact with both the catalytic and peripheral anionic sites of the enzyme, i.e., as dual binding site AChE inhibitors. Apart from inhibiting AChE, some of these compounds were also able to inhibit butyrylcholinesterase (BChE) and Aβ aggregation, thereby behaving as multitarget agents [11].

S. Zhang et al. present a study on the mechanisms of action that are behind the neuroprotective effects of a novel multitarget compound derived from the structural modification of a lead, which was developed by this group and combines a unit of the multifunctional compound curcumin and a cell membrane/lipid raft anchor steroidal moiety [12].

The replacement of the isosorbide-aryl-5-ester of a family of isosorbide-2-carbamates-5-aryl esters with potent and selective BChE inhibitory activity with a pharmacophoric moiety of the antioxidants ferulic or lipoic acid has led J. F. Gilmer et al. to develop hybrid compounds that retain a potent BChE inhibitory activity and display additional antioxidant and protective effects against the glutamate-induced neurotoxicity in HT-22 hippocampal cells [13].

A series of benzochromenepyrimidinone has been developed by L. Ismaili, J. Marco-Contelles, F. Chabchoub, et al. as nonhepatotoxic compounds endowed with human AChE inhibitory activity and antioxidant effects [14].

A. Rampa, F. Belluti, et al. describe a new family of multitarget compounds, which were designed by combination of a benzophenone-derived pharmacophoric moiety of a known BACE-1 inhibitor with a chalcone-derived metal chelating pharmacophore. The lead of the series displayed low micromolar BACE-1 inhibitory activity as well as protective effects against metal-associated oxidative stress [15].

M. L. Bolognesi, A. Cavalli, and F. Prati provide a critical overview on the needs and challenges to be faced in the rational design of hybrid- and fragment-like molecules purported to hit several key biological targets in the pathogenesis of AD by discussing some multitarget compounds developed in their group, which were designed to hit AChE, NMDA receptors, metal chelation, BACE-1, and GSK-3β [16].

Stimulation of neurogenesis has been recently proposed as a promising approach to treat neurodegenerative diseases. M. I. Rodríguez-Franco et al. review recent advances in the development of neurogenic agents, some of them designed as multitarget hybrid compounds [17].

Molecular hybridization has been used by Z. Li et al. to design a series of graveoline-based dual binding site inhibitors of AChE. The reported compounds exhibit nanomolar potency against AChE as well as micromolar BChE inhibitory activity, and one of them can inhibit the AChE-induced aggregation of Aβ42 [18].

A series of hybrids formally derived from donepezil and tacrine have been synthesized by I. Carvalho, A. Martínez, et al. as a novel family of dual binding site AChE inhibitors. These compounds, which feature an indanone ring system like donepezil, a quinoline moiety like tacrine and a triazole ring in the linker, have turned out to be micromolar inhibitors of AChE [19].

M. Krátký et al. describe a series of salicylanilide *N*,*N*-disubstituted carbamates and thiocarbamates as moderately potent dual inhibitors of AChE and BChE. Despite the presence of the carbamate moiety, typical of some active site inhibitors of AChE, these compounds seem to interact in great part at the peripheral sites of these enzymes without covalently modifying the catalytic serine residue [20].

Following previous reports on the positive effects of extracts of *Dipsacus asper* Wall. and one of its active principles, Akebia saponin D (ASD), on Aβ42-induced cognitive impairment, H. Ji, R. Hu, et al. describe the learning- and memory-improving effects of ASD in a rat model of sporadic AD, which seem to arise from modulation of the amyloidogenic pathway by down-regulation of the expression of BACE and presenilin 2 and from increased expression of TNFα converting enzyme (TACE), insulin-degrading enzyme (IDE), and lipoprotein receptor-related protein 1 (LRP-1) [21].

Apart from AChE and amyloid pathology, tau protein is another key molecular target for AD therapeutic and diagnostic agents. Y. Guan et al. describe the radiosynthesis and biological characterization of a novel [^11^C]-labeled tau imaging agent as a potential positron emission tomography (PET) radiotracer for AD diagnosis [22].

The activity of phospholipase A_2_ (PLA_2_), a key player in the regulation of the release from cell membranes of lipid mediators with important roles in signal transduction and regulation of inflammatory processes, is reduced in AD patients. O. V. Forlenza et al. report that long-term treatment with lithium increases the membrane phospholipid metabolism in cortical and hippocampal neurons by activation of total, cytosolic calcium-dependent, and calcium-independent PLA_2_, thereby constituting a potential option for the prevention of AD [23].

In light of the interrelationship and mutual reinforcement of the pathological mechanisms of AD and diabetes, which lead to an accelerated cognitive impairment in AD patients, insulin therapy is being considered for symptomatic AD treatment. S. Ribarič reviews the interactions between both diseases and describes the clinical results of intranasal administration of insulin for the treatment of cognitive impairment in AD patients [24].

Overall, this *Molecules* Special Issue gathers some recent advances in the development of novel drug candidates, diagnostic agents, and therapeutic strategies against AD, which evidence the huge and sustained research efforts and commitment of the scientific community in the battle against such a devastating disease.
